# The Surgeon's Practical Guide in Dressing, and in the Methodic Application of Bandages. Illustrated by Numerous Engravings

**Published:** 1835-01-01

**Authors:** 


*The Surgeon's Practical Guide in Dressing, and in the Methodic Application of Bandages. Illustrated by numerous Engravings.* By Thomas Cutler, m.d., late Staff-Surgeon in the Belgian Army.—London, 1834. 12mo. pp. 195.

In the present bookwriting days, it is a hard matter for an author to find an excuse for his work, or, at least, one with any share of novelty; and the consequence is, that the same hacknied sentence is transferred from volume to volume, without any examination as to its applicability to its new situation. Dr. Cutler commences his preface with the following statement:

"A pocket manual, calculated to improve a department of surgery which unfortunately has been too long neglected in this country, and spare the practitioner the sacrifice of much valuable time, cannot but be deemed a great desideratum."

Too much neglected in this country! Shade of Baynton, hear him! Where can Dr. Cutler have studied surgery, that he can venture on such an assertion? Has he never heard of Pott, Astley Cooper, Cline, and Amesbury? or has he so little acquaintance with the subject on which he writes, as to be ignorant of the various apparatus to which their names are affixed?

After reading his work, we readily acquit him of these charges, as he has not only very carefully described them, but attributed each to its respective inventor. It is quite clear, then, that our author never meant that this department of surgery was really neglected in this country; but he has adopted it as a convenient ready-made sentence, without examining whether it exactly fitted his subject. So far, indeed, is he from the truth, that we have no hesitation in affirming that there is no country where so much attention is paid to bandaging and dressing as in our own, and none in which they are brought to such great perfection. Nay, we will go further, and challenge Dr. Cutler to name one celebrated English surgeon who has not either invented or improved some mechanical apparatus.

We would not however quarrel with our author for seeking an excuse for his work: we grant that every author does, or ought to, think his book a desideratum; but we are not pleased that our country, and its reputation in the arts and sciences, should be libelled, in order to turn a neat sentence, or to form a graceful opening to a preface.

But to proceed to the work itself. When a surgeon undertakes to write upon a very confined subject, it is to be expected that the minuteness with which he enters upon it will serve as a substitute for more extended views. Accordingly, when a book with the title of the "Surgeon's Practical Guide in Dressing and in the Methodic Application of Bandages" was first placed in our hands, we were indeed disappointed to find little else but a *catalogue raisonnée* of a few surgical instruments and apparatus; not a single new invention, not even an improvement suggested by the author.

Dr. Cutler is evidently a disciple of the French school of surgery; he seems perfectly at home with their method of dressing and with their apparatus, but he does not appear to be equally well acquainted with the ordinary surgical machinery employed in his own country. For instance, in that portion of his work devoted to the means of reducing and retaining in due position fractures of the leg, we find no mention of the junks, one of the best and most common means of treatment. Again, in detailing the various kinds of trusses, he has omitted the spring truss in general use, the Maidstone truss, Egg's, and many others, and has contented himself with describing Salmon and Ody's, and Adam's graduated pressure trusses, which, though excellent in some cases, are open to several objections in others. In short, there is not a single portion of the work in which there are not gross omissions, even when looked upon in the humble light of a catalogue; but, when examined as a guide in the application of bandages, the descriptions are so short and superficial, that the reader, failing to trace even a resemblance to such a thing, turns to the title-page to ascertain if he has not misunderstood the author's intentions.

It would be injustice, however, to Dr. Cutler to close our notice of his book without mentioning the excellence of his drawings; to the description of each apparatus one or more illustrations is affixed, representing the mode of its application, and assisting the reader greatly in comprehending its previous parts and uses. These alone are worth the price of the whole.

We have thus stated our opinion freely of the beauties and errors of this work; we fully approve of its design, and, though we do not believe mechanical surgery to be neglected in this country, yet think a good manual a desideratum. Dr. Cutler appears, by his skill in drawing, and by the attention which he has paid to the bandages in use upon the continent, to be well fitted for publishing on the subject with success; but, ere he can expect to do so, he must spend some period in visiting the different hospitals of the metropolis, make himself acquainted with the various apparatus employed in each, with their relative advantages and disadvantages, and afterwards bestow a little more attention in describing the minutiæ of their application.

